# Optimizing processing methods for maximum bioactive retention: comparative metabolomic analysis of dried loquat (*Eriobotrya japonica*) flowers and their powdered extracts

**DOI:** 10.3389/fnut.2025.1637247

**Published:** 2025-08-01

**Authors:** Mingzheng Duan, Jieming Feng, Jing-Han Feng, Xi Wang, Xu Xiao, Shirong He, Hengcui Guo, Wenyan Zhang, Zhumao Jiang, Tongfa Wan, Muhammad Junaid Rao

**Affiliations:** ^1^Key Laboratory for Research on Zhaotong Apple Resources, College of Agronomy and Life Sciences, Zhaotong University, Zhaotong, China; ^2^Science and Technology Information Institute, Zhaotong Municipal Bureau of Science and Technology, Zhaotong, China; ^3^College of Chemistry and Chemical Engineering, Zhaotong University, Zhaotong, China; ^4^Yongshan County Bureau of Agriculture and Rural Affairs, Zhaotong, China; ^5^National Key Laboratory for Development and Utilization of Forest Food Resources, Zhejiang A&F University, Hangzhou, Zhejiang, China

**Keywords:** loquat flower flavonoids powder, freeze-drying vs. heat-drying, bioactive flavonoid compound preservation, UPLC–MS/MS metabolomics, antioxidant capacity

## Abstract

Loquat (*Eriobotrya japonica*) flowers are a rich source of bioactive flavonoids, but their nutraceutical potential depends on post-harvest processing. This study evaluated the impact of heat-drying (HD) and freeze-drying (FD) on flavonoid retention in loquat flowers and their hot-water powdered extracts using UPLC–MS/MS metabolomics and antioxidant assays. Freeze-drying significantly preserved thermolabile compounds, with cyanidin showing a 6.62-fold increase (Log2FC 2.73) in FD compared to HD, while delphinidin 3-O-beta-D-sambubioside surged 49.85-fold (Log2FC 5.64). In contrast, heat-drying degraded many flavonoids but selectively enhanced others, such as 6-hydroxyluteolin (27.36-fold increase, Log2FC 4.77), and methyl hesperidin showed highlest percentage abundance (10.03%). Freeze-dried powder (FDP) exhibited the highest antioxidant activity (608.83 μg TE/g), linked to elevated levels of key metabolites like eriodictyol chalcone (18.62-fold increase, Log2FC 4.22). Multivariate analyses confirmed distinct clustering, with FD samples closely grouped, indicating stable metabolite preservation. Heat-dried samples showed greater variability, reflecting thermal degradation and pathway activation. The results demonstrate that freeze-drying optimizes flavonoid retention, making it ideal for high-quality nutraceuticals, while heat-drying may suit cost-effective production of select heat-stable compounds. These insights guide the development of standardized loquat flower products, balancing bioactive preservation with processing efficiency for functional food and herbal medicine applications.

## 1 Introduction

Loquat (*Eriobotrya japonica* Lindl.) flowers, often overshadowed by the fruit, are a rich source of bioactive flavonoids with significant antioxidants and anti-inflammatory properties (1). These flowers have been traditionally used in herbal medicine, but their nutraceutical potential depends heavily on post-harvest processing (1, 2). Fresh plant materials are prone to enzymatic degradation, making drying essential for preservation. However, conventional methods like heat drying (HD) may degrade thermolabile compounds, while freeze-drying (FD) is known to better preserve heat-sensitive metabolites (3–5). Despite this general understanding, the specific impact of these drying techniques on loquat flower flavonoids (particularly when further processed into powdered extracts) remains unclear. This study systematically evaluates how different drying and extraction methods alter the phytochemical composition of loquat flowers, providing crucial insights for functional food and nutraceutical applications.

The choice of drying method plays a pivotal role in determining the final bioactive quality of plant-based products. Heat drying, though cost-effective, can lead to the degradation of flavonoids through thermal oxidation and Maillard reactions (3, 6, 7). In contrast, freeze-drying minimizes thermal damage, preserving structural integrity and bioactive content. However, most industrial applications involve additional processing steps, such as hot-water extraction and powder formation, which may further influence metabolite stability (8, 9). For instance, while freeze-dried flowers may retain more flavonoids initially, converting them into powdered extracts could introduce new variables affecting compound retention (5). This study not only compares fresh, heat-dried, and freeze-dried flowers but also examines their powdered derivatives to determine which processing chain best preserves key bioactive compounds.

Given the growing demand for natural bioactive compounds in functional foods, nutraceuticals, and herbal medicine, optimizing processing techniques to maximize flavonoid retention is crucial ((10–15)). Previous studies on other medicinal plants (e.g., *Moringa oleifera*, *Camellia sinensis*) have demonstrated that drying methods significantly influence antioxidant capacity and phenolic content (13, 16). However, loquat flowers present a unique case due to their complex flavonoid glycosides and anthocyanins, which may respond differently to thermal and lyophilization treatments (17–19). Understanding these variations is essential for industrial applications where bioactive consistency and potency are critical.

Flavonoids, the primary bioactive constituents in loquat flowers, contribute to their health benefits, including antioxidants, antidiabetic, and anti-inflammatory effects ((20–24)). Compounds like violanthin, cyanidin, and methyl hesperidin are particularly important antioxidant flavonoids (11, 23, 25–28), yet their stability under different processing conditions remains underexplored. Using UPLC–MS/MS metabolomics, this study identifies which flavonoids are most susceptible to degradation and which processing methods enhance or diminish their concentrations (29). Additionally, antioxidant activity assay correlates these compositional changes with functional properties, offering a practical perspective for product development (30–32). Understanding these dynamics is essential for optimizing processing techniques that maximize flavonoid retention while balancing economic feasibility.

Ultimately, this research bridges the gap between post-harvest processing and bioactive preservation in loquat flowers. By comparing fresh, heat-dried, and freeze-dried samples—along with their powdered extracts—we provide actionable insights for manufacturers aiming to produce high-quality loquat flower products. Whether for dietary supplements, functional foods, or herbal teas, the findings guide optimal drying and extraction protocols to ensure maximum retention of health-promoting flavonoids. This study not only advances scientific understanding of loquat flower metabolomics but also supports the growing demand for standardized, bioactive-rich natural products in the nutraceutical industry.

## 2 Materials and methods

### 2.1 Loquat flowers harvesting and laboratory processing

Loquat (*Eriobotrya japonica* cv. *Dawuxing*) flowers were harvested from Xiluodu Town, Yongshan County (103.64°E, 28.24°N), Zhaotong City, Yunnan Province, China, on November 25, 2024. Flowers were collected at the partially bloomed bud stage to ensure uniform maturity and physiological consistency. To minimize variability, samples were pooled from six healthy parent plants grown under identical environmental conditions. Collection was performed using sterilized scissors with careful handling to maintain structural integrity, and specimens were transported in sterile collection receptacles to prevent contamination and physical damage.

Laboratory processing involved an initial purification protocol wherein specimens underwent immersion in deionized water with gentle mechanical agitation to remove particulate contaminants, followed by moisture removal via sterile absorbent material and ambient air desiccation. The processed specimens were subsequently allocated into three experimental groups based on preservation methodology: refrigerated storage (4°C), thermal dehydration (60°C for 6 h until complete moisture removal), and lyophilization (preliminary freezing at −20°C followed by vacuum dehydration at −50°C for 48 h).

### 2.2 Extraction procedure

The extraction procedure employed thermal aqueous extraction at 90°C with 30-min duration using a standardized biomass-to-solvent ratio (1:20 w/v). Post-extraction, the solution underwent gravity separation for 6 h, facilitating supernatant isolation. The aqueous extract subsequently underwent lyophilization via preliminary freezing (−40°C) followed by vacuum dehydration (48 h), yielding a stable powdered extract amenable to long-term storage.

Quality control was maintained throughout the experimental procedure through utilization of precision instrumentation (temperature-controlled drying apparatus, lyophilization equipment, and ultra-low temperature storage) and implementation of aseptic techniques to preserve sample integrity. The resultant extracts (fresh, thermally dehydrated, and lyophilized) were hermetically sealed, appropriately labeled, and preserved for subsequent phytochemical analysis, particularly for flavonoid compound characterization and quantification.

### 2.3 Loquat flower flavonoid profiling

#### 2.3.1 Sample processing and metabolite isolation

Comprehensive metabolomic profiling of loquat fruit samples was conducted through ultra-performance liquid chromatography coupled with tandem mass spectrometry (UPLC-MS/MS) in collaboration with Wuhan Metware Biotechnology Co., Ltd., Wuhan, China,^1^ following established protocols ((33)). Loquat flower samples underwent vacuum freeze-drying treatment using a Scientz-100F lyophilizer for a duration of 63 h. The lyophilized material was subsequently ground to a fine powder using a ball mill apparatus (MM 400, Retsch) at 30 Hz for 1.5 min.

For each analysis, 30 mg of the powdered sample was accurately weighed using a precision balance (MS105DΜ) and placed in extraction vessels. Metabolite isolation was achieved by adding 1,500 μL of pre-cooled (−20°C) 70% methanol–water solution containing internal standards (2-chlorophenylalanine at 1 mg/L concentration), maintaining a sample-to-solvent ratio of 1:50 (w/v). The samples underwent periodic vortex agitation (30 s at 30-min intervals) for six cycles to ensure thorough extraction. Subsequently, samples were centrifuged using an Eppendorf Model 5424R centrifuge at 12,000 rpm for 3 min, and the supernatant was collected. The extract was filtered through 0.22 μm membrane filters and transferred to autosampler vials for UPLC–MS/MS analysis.

#### 2.3.2 Chromatographic separation conditions

Analytical separation was accomplished using an integrated UPLC-ESI-MS/MS system featuring an ExionLC™ AD ultra-performance liquid chromatography instrument^2^ interfaced with a triple quadrupole mass spectrometer (see footnote 2). Compound separation was achieved on an Agilent SB-C18 analytical column (1.8 μm, 2.1 mm × 100 mm). The mobile phase consisted of solvent A (ultrapure water with 0.1% formic acid) and solvent B (acetonitrile containing 0.1% formic acid). The gradient program began with 95% A and 5% B, transitioning linearly to 5% A and 95% B over 9 min. This composition was held for 1 min, then rapidly returned to starting conditions (95% A, 5% B) within 1.1 min, followed by a 2.9-min equilibration period. Operating parameters included a flow rate of 0.35 mL/min, column temperature of 40°C, and injection volume of 2 μL. The column eluent was directed to an electrospray ionization triple quadrupole linear ion trap mass spectrometer (ESI-QTRAP-MS).

#### 2.3.3 Mass spectrometric operating conditions

Mass spectrometric detection was performed using an ESI-QTRAP-MS system with optimized parameters as follows: ion source temperature of 500°C; electrospray voltages of +5,500 V (positive mode) and −4,500 V (negative mode); nebulizer gas (GS1), auxiliary gas (GS2), and curtain gas pressures of 50, 60, and 25 psi, respectively; collision-activated dissociation gas pressure set to high. Multiple reaction monitoring (MRM) experiments were conducted in triple quadrupole mode using nitrogen as collision gas at medium pressure. Individual optimization of declustering potential (DP) and collision energy (CE) parameters was performed for each MRM transition to achieve optimal sensitivity and selectivity. Time-segmented MRM data acquisition was implemented, monitoring specific transition sets within predetermined retention time windows that corresponded to target metabolite elution profiles, as previously described (33). Metabolite identification was accomplished by matching accurate ion pairs, precursor ion (Q1) and product ion (Q3) values, and fragmentation patterns against reference databases (complete metabolic data available in Supplementary Table S1).

### 2.4 Evaluation of free radical scavenging capacity in loquat using DPPH assay

The antioxidant capacity of variously processed loquat flower preparations was assessed using the 2,2-diphenyl-1-picrylhydrazyl (DPPH) free radical scavenging assay (34). The samples analyzed included fresh flowers, freeze-dried specimens, oven-dried specimens, and their corresponding hot-water-extracted powders.

Sample preparation involved homogenizing 0.1 g of each specimen in 1 mL of 80% methanol. The mixtures underwent ultrasonic-assisted extraction at 60°C for a 30-min period with periodic agitation, followed by centrifugation at 12,000 rpm to harvest the supernatant. The analytical procedure consisted of combining equal volumes (150 μL) of the extracted supernatant and DPPH working solution (prepared in ethanol) in microtubes, while the control preparation contained only methanol. Following a 30-min dark incubation period, spectrophotometric measurements were taken at 517 nm wavelength using a microplate reader. Antioxidant potency was expressed as micro-gram Trolox equivalents per gram of sample (μg TE/g) through quantification against a standard curve prepared with Trolox concentrations ranging from 0 to 25 μg/mL.

### 2.5 Clustering and statistical analysis

Multivariate analytical approaches were employed to examine flavonoid and anthocyanin distribution patterns alongside their corresponding gene expression profiles. Prior to analysis, both metabolomic and transcriptomic datasets underwent Z-score normalization. To distinguish between experimental groups while reducing intra-group variability, hierarchical cluster analysis (HCA) and principal component analysis (PCA) were conducted using OmicShare tools, an open-access online analytical platform (35).^4^ Visual presentations and standard error computations were prepared using Microsoft Excel (Redmond, WA, USA). For statistical evaluation of biochemical parameters, Statistix 8.1 software (Tallahassee, FL, USA) was employed, with all experimental procedures performed in triplicate. The least significant difference (LSD) test was applied to assess statistical significance of variations in antioxidant activity and total flavonoid and anthocyanin concentrations across loquat samples.

Variable importance in projection (VIP) values were determined using orthogonal partial least squares-discriminant analysis via the MetaboAnalystR package within the R statistical environment (61). Metabolites exhibiting VIP values above 1.0 were classified as significant discriminatory biomarkers for distinguishing experimental groups (62). The selection of significantly modified metabolites followed a comprehensive dual-criterion approach: statistical significance was assessed using Student’s t-test with false discovery rate adjustment (*p* < 0.05) (63), while biological significance was determined through fold-change thresholds (|log2FC| ≥ 1, corresponding to FC ≥ 2 or ≤ 0.5) (64). Venn diagram visualization was performed using the EVenn web-based tool (35).^5^

## 3 Results

### 3.1 Differential metabolites changes in different processed loquat flower groups

**Figure 1:**
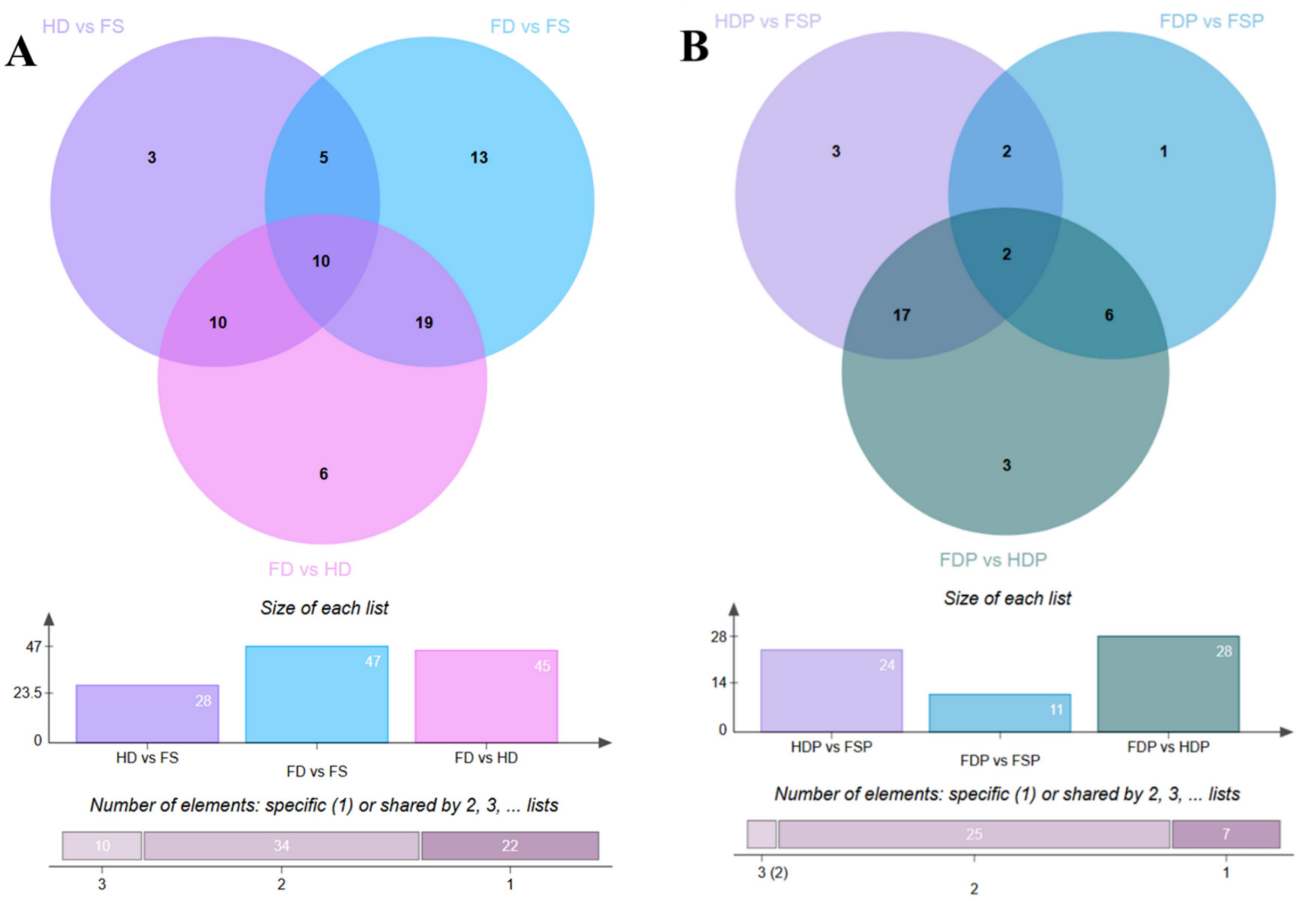
Venn diagrams illustrate shared and unique compounds in loquat flower samples. **(A)** Comparison of raw sample groups (HD, FD, FS). **(B)** Comparison of processed sample groups (HDP, FDP, FSP). FS, fresh sample; HD, heat drying; FD, freeze-drying; FSP, fresh sample powder; HDP, heat drying powder; FDP, freeze-drying powder.

Venn diagram analysis of loquat flower metabolites revealed distinct compositional differences between sample groups. In different samples, 10 compounds were common across all groups, while pairwise comparisons showed unique metabolites: HD vs. FS had 3, FD vs. FS had 13, and FD vs. HD had 6 (Figure 1A). The total number of significantly altered compounds was highest in FD vs. FS (47), followed by FD vs. HD (45) and HD vs. FS (28), suggesting that processing methods significantly influence metabolite profiles. These variations may stem from differences in enzymatic activity, thermal degradation, or extraction efficiency, highlighting the importance of processing conditions in preserving bioactive compounds.

A similar trend was observed in processed samples, where 10 shared compounds were detected (Figure 1B). However, fewer unique metabolites were identified: HDP vs. FSP had 3, FDP vs. FSP had only 1, and FDP vs. HDP had 3 (Figure 1B). The total altered compounds were highest in FDP vs. HDP (28), followed by HDP vs. FSP (24) and FDP vs. FSP (11). This reduction in unique compounds compared to raw samples suggests that processing may homogenize certain metabolites, possibly due to thermal degradation or chemical interactions. These findings provide valuable insights into how different treatments affect loquat flower chemistry, which could guide optimal processing methods for retaining functional compounds in food applications.

**Figure 2:**
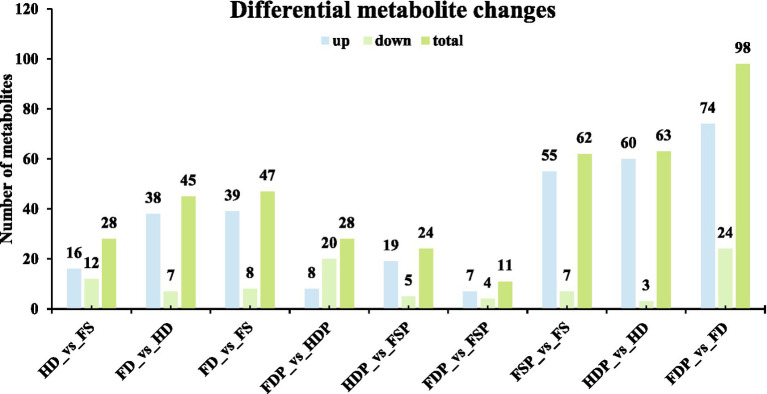
Differential metabolite changes in loquat flower samples under varying processing conditions. Column graphs depict the number of upregulated (light blue), downregulated (pale red), and total (light green) compounds in different pairwise comparison groups. Each bar indicates biological replicates (*n* = 3).

The comparative metabolomic analysis of loquat flower samples revealed significant variations in metabolite composition across different processing and storage conditions (Figure 2). In raw samples, the FD (freeze-dried) vs. FS (fresh) comparison exhibited the highest number of upregulated compounds (39 out of 47), suggesting that freeze-drying better preserves or even concentrates certain metabolites compared to fresh samples (Figure 2). Conversely, HD (heat-dried) samples showed fewer upregulated compounds (16 in HD vs. FS), implying potential thermal degradation (Figure 2). Interestingly, FD vs. HD had 38 upregulated metabolites, reinforcing that freeze-drying retains more bioactive compounds than heat-drying.

Processed samples displayed contrasting trends: FDP (freeze-dried powder) vs. HDP (heat-dried powder) had more downregulated compounds (20 vs. 8-upregulated). Notably, FDP vs. FD showed a dramatic shift, with 74 upregulated and 24 downregulated metabolites—the highest overall change (98 total), indicating that freeze-drying powder drastically alters the metabolic profile. Meanwhile, HDP vs. HD had 60 upregulated and only 3 downregulated compounds, suggesting heat-dried powder extract stabilizes certain metabolites. These findings highlight that processing methods—particularly fermentation and drying techniques—profoundly influence loquat flower chemistry, with implications for optimizing functional food production.

### 3.2 Hierarchical cluster analysis of loquat flower metabolites

**Figure 3:**
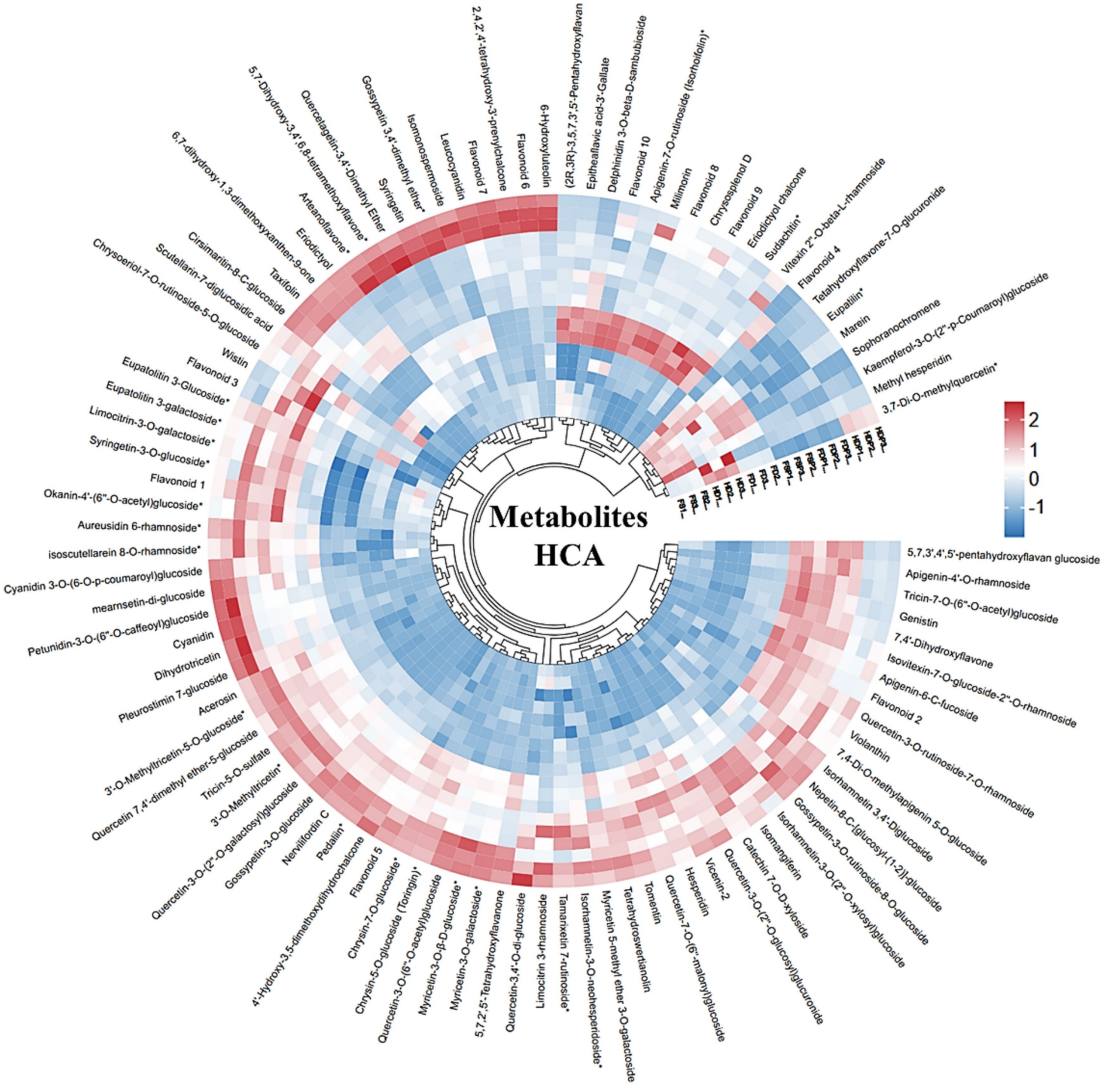
Hierarchical cluster analysis (HCA) of loquat flower metabolites across processing methods. Dendrogram and heatmap display metabolite abundance (row were normalized) in fresh (FS and FSP), heat-dried (HD, HDP), and freeze-dried (FD, FDP) samples. Flavonoid 1–10 names are mentioned in Supplementary Table S2. Color intensity reflects relative concentrations (red: high; blue: low). Clusters highlight distinct metabolite profiles driven by processing. FS, fresh sample; HD, heat drying; FD, freeze-drying; FSP, fresh sample powder; HDP, heat drying powder; FDP, freeze-drying powder.

Hierarchical cluster analysis (HCA) of loquat flower metabolites revealed distinct grouping patterns based on processing methods (Figure 3). HCA analysis revealed that the freeze-dried samples (FD, FDP) exhibited the closest similarity, suggesting that freeze-drying preserves metabolite profiles more consistently than heat treatments. In contrast, heat-dried samples (HD, HDP) clustered distinctly, likely due to thermal degradation of heat-sensitive compounds such as flavonoid glycosides. Fresh samples (FS and FSP) formed a separate group, indicating that drying—regardless of method—significantly alters the metabolite composition compared to untreated flowers.

Within clusters, powdered extracts (FSP, HDP, FDP) showed tighter grouping than their non-powdered counterparts, suggesting that the hot-water extraction and freeze-drying process homogenizes certain metabolites (Figure 3). For example, violanthin and syringetin-3-O-glucoside were highly abundant in freeze-dried powders (FDP), while 5,7,2′,5′-tetrahydroxyflavanone and quercetin derivatives dominated fresh samples. Heat-dried powders (HDP) showed reduced levels of thermally labile compounds like myricetin glycosides but retained higher concentrations of heat-stable metabolites such as tricin derivatives (Figure 3). These findings underscore that processing methods—particularly drying temperature and extraction—profoundly influence loquat flower chemistry, with freeze-drying better preserving bioactive flavonoids.

### 3.3 Principal component analysis of loquat flower metabolites

**Figure 4:**
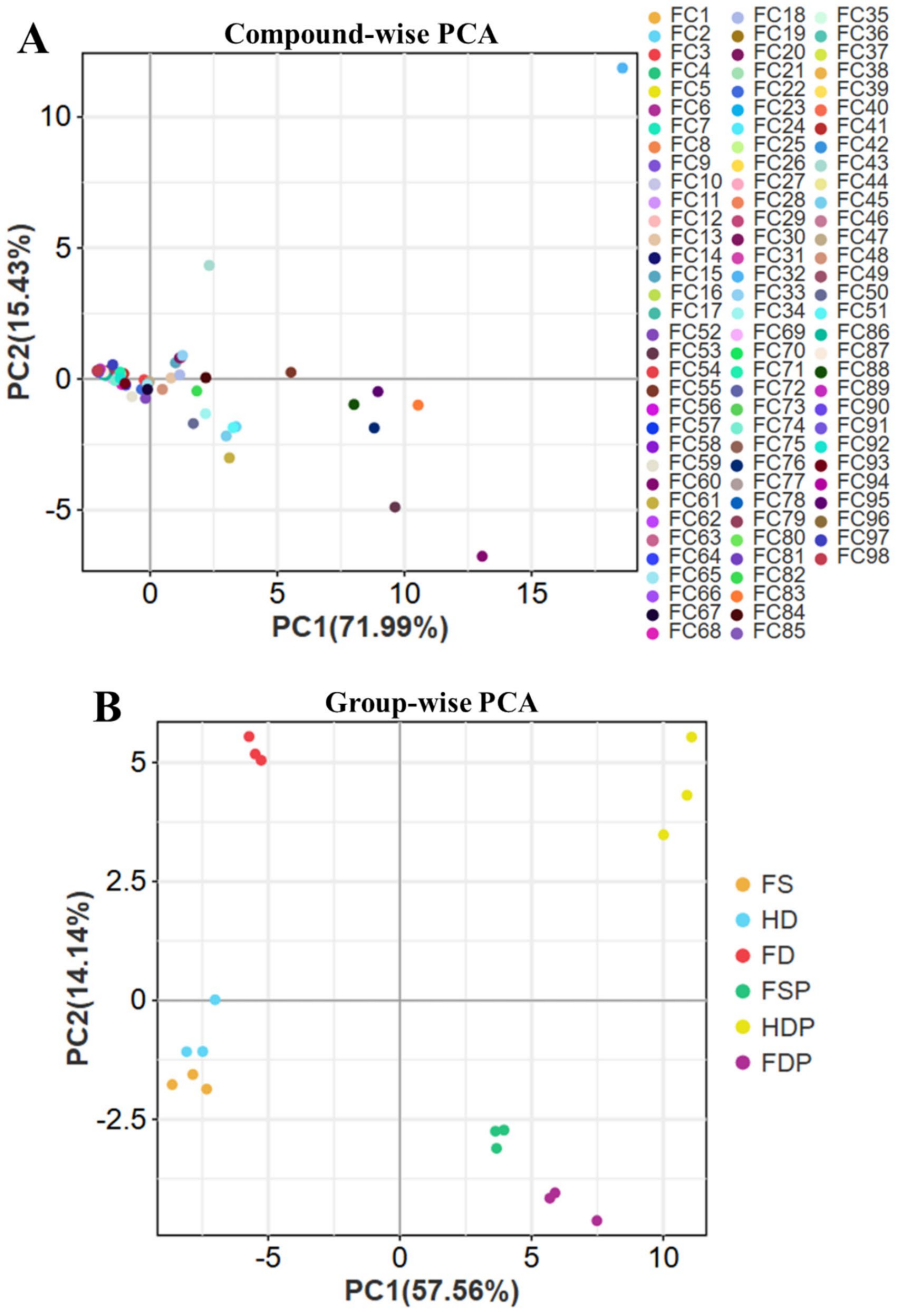
Principal component analysis (PCA) of loquat flower metabolites across processing methods. **(A)** Compound-wise PCA (PC1: 71.99%, PC2: 15.43%). FC1-98 names denote individual flavonoid compound represented in Supplementary Table S2. **(B)** Group-wise PCA (PC1: 57.56%, PC2: 14.14%) highlighting processing-based separation. FS, fresh sample; HD, heat drying; FD, freeze-drying; FSP, fresh sample powder; HDP, heat drying powder; FDP, freeze-drying powder.

Principal component analysis (PCA) of loquat flower metabolites revealed distinct clustering patterns based on processing methods (Figures 4A,B). In compound-wise PCA (Figure 4A, PC1: 71.99%, PC2: 15.43%), most compounds clustered tightly along the x-axis and y-axis intersection point, indicating high metabolite consistency (Figure 4A). Fresh samples (FS and FSP) formed a separate group, highlighting significant compositional changes induced by drying. Key compounds like violanthin (FC2) and syringetin-3-O-glucoside (FC29) heavily influenced FD/FDP clustering, underscoring their stability under freeze-drying. In contrast, heat-sensitive flavonoids (e.g., myricetin glycosides (Myricetin-3-O-*β*-D-glucoside - FC16)) were reduced in HD/HDP, aligning with PC2’s separation of thermally processed samples. Notably, FC12 (Okanin-4′-(6”-O-acetyl)glucoside) and FC55 (Cyanidin 3-O-(6-O-p-coumaroyl)glucoside) were pivotal in differentiating FD/FDP, correlating with their preservation of antioxidant polyphenols.

The group-wise PCA (Figure 4B, PC1: 57.56%, PC2: 14.14%) further emphasized processing-driven divergence (Figure 4B). Powdered extracts (FSP, HDP, FDP) clustered separately than their non-powdered counterparts (FS, HD, and FD), suggesting that hot-water extraction and freeze-drying extraction procedure significantly altered the metabolic compositions (Figure 4B). PC1 clearly separated freeze-dried from heat-dried groups, while PC2 distinguished fresh samples, likely due to retained volatiles and unaltered glycosides. These results demonstrate that freeze-drying better maintains bioactive integrity, while heat processing introduces variability, critical for optimizing loquat flower-based functional foods.

### 3.4 Key bioactive metabolites differentiated among groups (% peak area)

**Figure 5:**
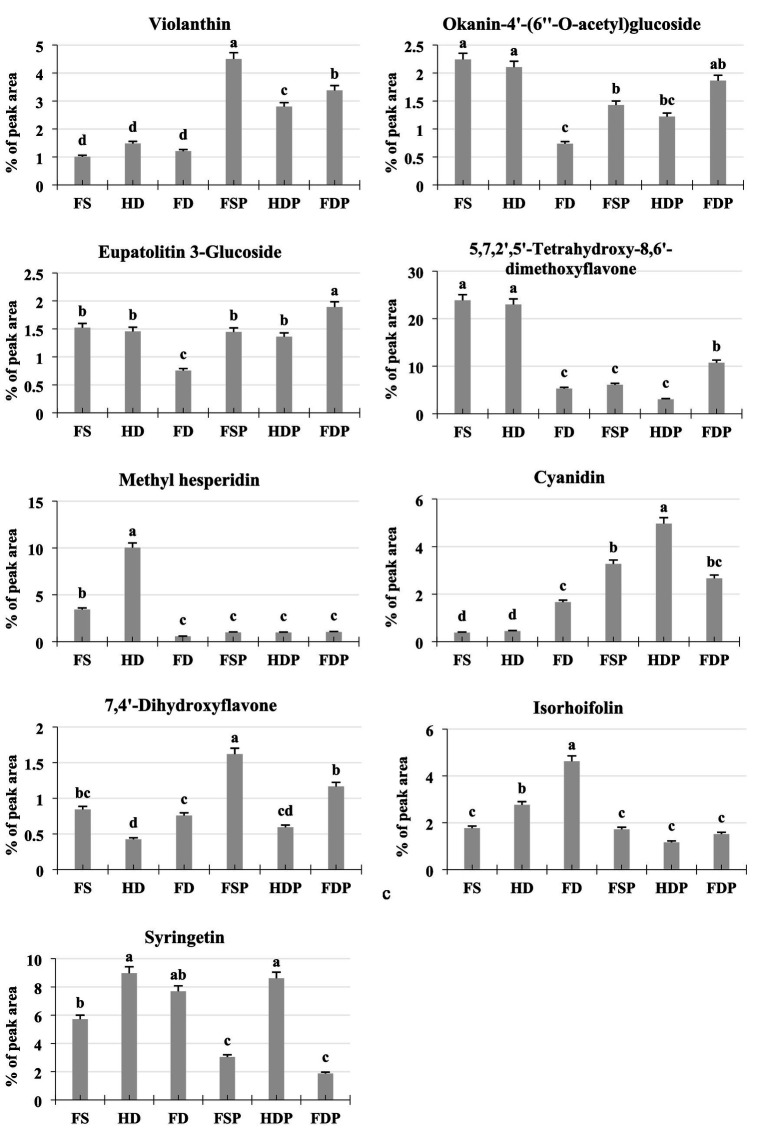
Concentration variations of key bioactive metabolites in loquat flowers under different processing methods. Column graphs show relative percentage peak area of eight representative metabolites (violanthin, okanin-4′-acetylglucoside, eupatolitin-3-glucoside, 5,7,2′,5′-tetrahydroxy-8,6′-dimethoxyflavone, methyl hesperidin, cyanidin, 7,4′-dihydroxyflavone, apigenin-7-O-rutinoside, and syringetin) across fresh sample (FS), heat drying (HD), freeze-drying (FD), fresh sample powder (FSP), heat drying powder (HDP), and freeze-drying powder (FDP). Values represent means ± standard deviation (*n* = 3) generated from LC-MS/MS data. Different lowercase letters indicate significant differences between groups (*p* < 0.05 by LSD test).

The analysis of key bioactive metabolites in loquat flower samples revealed significant variations influenced by processing methods (Figure 5). Violanthin, a major flavonoid, showed the highest percentage abundance in hot-water powdered extract samples, with FSP (4.50%) > FDP (3.38%) > HDP (2.80%), suggesting that extraction and powdering increased compound concentration. Interestingly, fresh samples contained moderate levels (FS:1.01%), while freeze-drying (FD:1.21%) better preserved violanthin compared to heat-drying (HD:1.48%) (Figure 5). Okanin and eupatolitin derivatives showed moderate processing effects, with powdered forms generally having higher concentrations than their non-powdered counterparts, likely due to concentration during extraction (Figure 5). For methyl hesperidin, heat-dried samples (HD:10.04) contained dramatically higher levels than other samples, likely due to thermal-induced conversion of precursors, while freeze-drying (FD:0.57%) and powdered forms showed reduced amounts (Figure 5). Cyanidin, an important anthocyanin, exhibited an opposite trend, with freeze-dried samples (FD:1.66%) and powders (FSP:3.27%, FDP:2.67%) containing substantially higher levels than fresh (FS:0.38%) or heat-dried (HD:0.45%) samples, suggesting freeze-drying better preserves this heat-sensitive pigment (Figure 5).

Notably, syringetin and apigenin-7-O-rutinoside showed complex patterns. Syringetin was abundant across all samples but highest in heat-dried (HD:8.98%) and powdered heat-dried (HDP:8.61%) forms, indicating thermal stability. The flavone 5,7,2′,5′-tetrahydroxy-8,6′-dimethoxyflavone demonstrated dramatic processing effects, with fresh samples (FS:23.85%) and head-dried (HD:22.99%) containing 4–5 times more than freeze-dried (FD:5.29) or powdered samples, suggesting significant degradation during processing (Figure 5). These findings highlight that processing methods differentially affect various bioactive classes—while some compounds like violanthin and cyanidin are preserved or enhanced in powdered freeze-dried forms, others like methyl hesperidin and certain flavones are either heat-converted or degraded.

### 3.5 Comparative metabolomic analysis of loquat flowers subjected to different drying methods

The metabolomic profiling of loquat flowers revealed significant variations in bioactive compounds depending on the drying method. Comparative analysis revealed FD significantly enhanced flavonoid content relative to both HD and FS samples (Figure 2), with 24 and 23 compounds showing >5-fold increases in FD vs. HD and FD vs. FS comparisons, respectively (Table 1). Freeze-drying markedly increased the abundance of key flavonoids, including delphinidin 3-O-beta-D-sambubioside (49.85-fold vs. HD), epitheaflavic acid-3′-gallate (23.35-fold vs. HD), gossypetin 3,4′-dimethyl ether (21.09-fold vs. HD), and eriodictyol chalcone (18.6-fold vs. FS), while heat-drying caused significant losses in glycosylated compounds (Table 1). Notably, cyanidin, an anthocyanin critical for color and health benefits, was 6.62-fold higher in FD than HD, confirming freeze-drying’s superiority in preserving thermolabile pigments (Table 1). Meanwhile, FD vs. FS comparisons indicated that freeze-drying not only preserves but may even enhance certain metabolites, such as pedaliin (18.01-fold increase) and eriodictyol chalcone (18.62-fold increase), likely due to the inhibition of enzymatic degradation during processing (Table 1). In contrast, heat drying led to the degradation of certain thermolabile compounds in comparison with FD, including methyl hesperidin (−3.31 Log2FC), eriodictyol (−2.34 Log2FC), taxifolin (−1.72 Log2FC) and scutellarin-7-diglucosidic acid (−1.81 Log2FC), suggesting that high temperatures may compromise flavonoid stability (Supplementary Table S3).

**Table 1:** Differential flavonoid metabolites in loquat flowers (FD: freeze-dried, HD: heat-dried, FS: fresh samples) identified by UPLC-MS/MS.

Serial No.	Compounds	FD vs HD				
		VIP	*p*-value	Fold change	Log2FC	Type
1	Delphinidin 3-O-beta-D-sambubioside	1.10	0.000	49.85	5.64	Up
2	Epitheaflavic acid-3’-Gallate	1.17	0.001	23.35	4.55	Up
3	Gossypetin 3,4′-dimethyl ether	1.16	0.015	21.09	4.40	Up
4	Eriodictyol chalcone	1.16	0.012	18.6	4.22	Up
5	Vitexin 2”-O-beta-L-rhamnoside	1.16	0.015	14.28	3.84	Up
6	Catechin 7-O-D-xyloside	1.15	0.012	9.19	3.20	Up
7	Acerosin	1.16	0.000	7.23	2.85	Up
8	(2R,3R)-3,5,7,3′,5’-Pentahydroxyflavan	1.16	0.003	7.08	2.82	Up
9	Cyanidin	1.15	0.022	6.62	2.73	Up
10	Apigenin-4’-O-rhamnoside	1.16	0.001	6.43	2.68	Up
11	Tamarixetin 7-rutinoside	1.15	0.009	6.40	2.68	Up
12	Nervilifordin C	1.16	0.016	6.25	2.64	Up
13	Tricin-7-O-(6”-O-acetyl)glucoside	1.16	0.003	6.19	2.63	Up
14	5,7,2′,5’-Tetrahydroxyflavanone	1.16	0.012	6.15	2.62	up
15	Quercetin-3-O-(2”-O-galactosyl)glucoside	1.16	0.011	6.05	2.60	Up
16	Quercetin 7,4′-dimethyl ether-5-glucoside	1.16	0.009	5.93	2.57	Up
17	Quercetin-3-O-(2”-O-glucosyl)glucuronide	1.16	0.013	5.86	2.55	Up
18	Gossypetin-3-O-glucoside	1.16	0.008	5.85	2.55	Up
19	Tomentin	1.16	0.006	5.76	2.53	Up
20	Tricin-5-O-sulfate	1.16	0.009	5.71	2.51	Up
21	Vicenin-2	1.16	0.013	5.62	2.49	Up
22	Isoscutellarein 8-O-rhamnoside	1.16	0.006	5.38	2.43	Up
23	3’-O-Methyltricetin	1.17	0.002	5.34	2.42	Up
24	5,8,3′-Trihydroxy-6,7,4′-trimethoxyflavone-8-O-β-D-glucoside	1.17	0.001	5.17	2.37	Up

Serial No.	Compounds	FD vs FS				
		VIP	*p*-value	Fold change	Log2FC	Type
1	Eriodictyol chalcone	1.21	0.012	18.62	4.22	Up
2	Pedaliin	1.21	0.007	18.01	4.17	Up
3	Vitexin 2”-O-beta-L-rhamnoside	1.21	0.015	14.3	3.84	Up
4	Catechin 7-O-D-xyloside	1.20	0.012	13.16	3.72	Up
5	Sudachitin	1.20	0.000	9.07	3.18	Up
6	Chrysosplenol D	1.21	0.003	8.99	3.17	Up
7	2-(3,4-Dimethoxyphenyl)-3,5,7-trihydroxy-8-methoxychromen-4-one	1.21	0.003	8.10	3.02	Up
8	5,7,4′-Trihydroxy-6,3′,5′-trimethoxyisoflavone	1.21	0.000	7.82	2.97	Up
9	Wistin	1.21	0.004	7.43	2.89	Up
10	4’-Hydroxy-3,5-dimethoxydihydrochalcone	1.21	0.009	6.93	2.79	Up
11	Cyanidin	1.20	0.022	6.62	2.73	Up
12	6-Hydroxyluteolin	1.19	0.047	6.27	2.65	Up
13	Nervilifordin C	1.20	0.016	6.25	2.64	Up
14	5,7,2′,5’-Tetrahydroxyflavanone	1.21	0.012	6.15	2.62	Up
15	Quercetin-3-O-(2”-O-galactosyl)glucoside	1.21	0.011	6.05	2.60	Up
16	Quercetin 7,4′-dimethyl ether-5-glucoside	1.21	0.009	5.93	2.57	Up
17	Quercetin-3-O-(2”-O-glucosyl)glucuronide	1.21	0.013	5.86	2.55	Up
18	Gossypetin-3-O-glucoside	1.21	0.008	5.85	2.55	Up
19	Chrysoeriol-7-O-rutinoside-5-O-glucoside	1.21	0.004	5.76	2.53	Up
20	Tomentin	1.21	0.006	5.76	2.53	Up
21	Vicenin-2	1.21	0.013	5.62	2.49	Up
22	3’-O-Methyltricetin	1.21	0.002	5.34	2.42	Up
23	5,8,3′-Trihydroxy-6,7,4′-trimethoxyflavone-8-O-β-D-glucoside	1.21	0.001	5.17	2.37	Up

Interestingly, HD vs. FS results showed a mixed response (Figure 2): while some compounds like 6-hydroxyluteolin (27.36-fold increase) and eriodictyol (11.72-fold increase) were significantly elevated, others, including tricin-7-O-(6”-O-acetyl)glucoside and gossypetin 3,4′-dimethyl ether, were markedly reduced (Supplementary Table S3). This suggests that heat drying may induce both thermal degradation and potential thermal activation of certain metabolic pathways. Overall, freeze-drying appears to be the most effective method for preserving bioactive flavonoids, whereas heat drying may be suitable for extracting specific compounds but risks losing others.

#### 3.5.1 Impact of drying methods on bioactive compounds in loquat flower powder and extracts

The comparative metabolomic analysis of loquat flower powders (FDP: freeze-dried powder, HDP: heat-dried powder, FSP: fresh sample powder) hot-water extracts revealed significant variations in flavonoid and phenolic content (Supplementary Table S4). When comparing FDP vs. HDP, freeze-drying led to the upregulation of key compounds such as delphinidin 3-O-beta-D-sambubioside (3.14-fold increase), 5,7,2′,5′-tetrahydroxy-8,6′-dimethoxyflavone (2.88-fold increase), 5,7,3′,4′,5′-pentahydroxyflavan glucoside (2.54-fold increase) and tricin-7-O-(6”-O-acetyl)glucoside (2.27-fold increase), while several others, including 5,7,2′,5′-tetrahydroxyflavanone (−1.67 Log2FC) and 6-hydroxyluteolin (−2.76 Log2FC), were significantly reduced (Table 2). Notably, sophoranochromene exhibited a striking 6.04-fold increase in FDP vs. HDP, suggesting freeze-drying may enhance certain unique metabolites (Table 2). Conversely, heat-dried powder (HDP vs. FSP) showed a contrasting trend, with 6-hydroxyluteolin (4.59-fold increase) and quercetagetin-3,4′-dimethyl ether (5.14-fold increase) being markedly elevated, while delphinidin 3-O-beta-D-sambubioside (0.16-fold decrease) and apigenin-4’-O-rhamnoside (0.46-fold decrease) were suppressed (Supplementary Table S4). These findings indicate that heat drying may promote the stability of some flavonoids while degrading others, possibly due to thermal modifications or oxidative reactions.

**Table 2:** Comparative analysis of key bioactive metabolites in loquat flower hot-water extracts under different processing methods.

Serial No.	Compounds	FDP vs HDP				
		VIP	*p*-value	Fold change	Log2FC	Type
1	Sophoranochromene	1.23	0.03	6.04	2.59	Up
2	Delphinidin 3-O-beta-D-sambubioside	1.21	0.01	3.14	1.65	Up
3	5,7,2′,5’-Tetrahydroxy-8,6′-dimethoxyflavone	1.24	0.00	2.88	1.52	Up
4	5,7,3′,4′,5′-Pentahydroxyflavan glucoside	1.24	0.00	2.54	1.34	Up
5	Wistin	1.11	0.04	2.31	1.21	Up
6	Tricin-7-O-(6”-O-acetyl)glucoside	1.20	0.01	2.27	1.18	Up
7	Apigenin-4’-O-rhamnoside	1.22	0.02	2.25	1.17	Up
8	Isorhamnetin-3-O-(6″-malonyl)glucoside-7-O-glucoside	1.01	0.04	2.00	1.00	Up
9	Leucocyanidin	1.24	0.00	0.49	−1.02	Down
10	Petunidin-3-O-(6”-O-caffeoyl)glucoside	1.16	0.06	0.49	−1.03	Down
11	Dihydrotricetin	1.14	0.05	0.48	−1.05	Down
12	Marein	1.24	0.00	0.48	−1.06	Down
13	Cyanidin	1.20	0.03	0.44	−1.20	Down
14	(2r,3 s)-2-(2,4,5-Trihydroxyphenyl)-3,4-dihydro-2 h-1-benzopyran-3,5,7-triol	1.24	0.00	0.41	−1.30	Down
15	Myricetin-3-O-β-D-glucoside	1.23	0.01	0.41	−1.30	Down
16	3,7-Di-O-methylquercetin	1.24	0.00	0.35	−1.52	Down
17	2,4,2′,4′-Tetrahydroxy-3′-prenylchalcone	1.24	0.00	0.34	−1.55	Down
18	5,7,2′,5’-Tetrahydroxyflavanone	1.24	0.00	0.31	−1.67	Down
19	6,7-Dihydroxy-1,3-dimethoxyxanthen-9-one	1.24	0.00	0.31	−1.71	Down
20	Eriodictyol	1.24	0.00	0.30	−1.75	Down
21	5,8,3′-Trihydroxy-6,7,4′-trimethoxyflavone-8-O-β-D-glucoside	1.24	0.00	0.28	−1.82	Down
22	5,7-Dihydroxy-3,4′,6,8-tetramethoxyflavone	1.22	0.00	0.22	−2.20	Down
23	5,7-Dihydroxy-6,3′,4′,5′-tetramethoxyflavone (Arteanoflavone)	1.23	0.01	0.22	−2.22	Down
24	Isomonospermoside	1.24	0.01	0.21	−2.25	Down
25	Syringetin	1.25	0.00	0.18	−2.50	Down
26	Gossypetin 3,4′-dimethyl ether	1.24	0.01	0.16	−2.67	Down
27	6-Hydroxyluteolin	1.23	0.00	0.15	−2.76	Down
28	Quercetagetin-3,4’-Dimethyl Ether	1.24	0.02	0.14	−2.79	Down

Serial No.	Compounds	FDP vs FSP				
		VIP	*p*-value	Fold change	Log2FC	Type
29	Isomonospermoside	1.41	0.00	6.59	2.72	Up
30	Sophoranochromene	1.39	0.03	6.04	2.59	Up
31	Limocitrin 3-rhamnoside	1.26	0.11	2.39	1.25	Up
32	Scutellarin-7-diglucosidic acid	1.33	0.01	2.35	1.23	Up
33	Wistin	1.22	0.04	2.20	1.14	Up
34	Isomangiferin	1.40	0.00	2.16	1.11	Up
35	5,7,2′,5’-Tetrahydroxy-8,6′-dimethoxyflavone	1.39	0.00	2.06	1.04	Up
36	Delphinidin 3-O-beta-D-sambubioside	1.37	0.001	0.49	−1.01	Down
37	Eriodictyol	1.40	0.0001	0.42	−1.24	Down
38	6,7-Dihydroxy-1,3-dimethoxyxanthen-9-one	1.40	0.0009	0.42	−1.25	Down
39	5,7,2′,5’-Tetrahydroxyflavanone	1.39	0.009	0.417	−1.25	Down

The table highlights significant changes in compounds when comparing freeze-dried powder (FDP) to heat-dried powder (HDP) and fresh sample powder (FSP). Values represent fold changes, VIP scores (Variable Importance in Projection), and statistical significance (*p*-value). Upregulated compounds (positive Log2FC) indicate higher retention or concentration in FDP, while downregulated compounds (negative Log2FC) suggest degradation or reduced extractability.

The FDP vs. FSP comparison demonstrated that freeze-drying preserves certain compounds more effectively than fresh processing. For instance, limocitrin 3-rhamnoside (2.39-fold increase), isomangiferin (2.16-fold increase), and scutellarin-7-diglucosidic acid (2.35-fold increase) were retained at higher levels in FDP, whereas 5,7,2′,5’-Tetrahydroxyflavanone (−1.25 Log2FC down). eriodictyol (−1.24 Log2FC down) and delphinidin 3-O-beta-D-sambubioside (−1.01 Log2FC down) were reduced (Table 2). Interestingly, isomonospermoside and sophoranochromene showed a dramatic 6.59- and 6.05-fold increase in FDP respectively, suggesting that freeze-drying may either concentrate these compounds or prevent their degradation (Table 2). These results highlight that freeze-drying is particularly effective for stabilizing heat-sensitive flavonoids, though some compounds may still undergo changes due to dehydration effects.

#### 3.5.2 Drying methods dramatically alter the phytochemical profile of loquat flowers

The metabolomic analysis of loquat flower extracts revealed profound differences in flavonoid and phenolic content. Freeze-dried powder (FDP) extract exhibited superior retention of bioactive compounds compared to fresh samples, with 5,7,2′,5′-tetrahydroxyflavanone (58.1-fold increase in FSP vs. FS) and quercetin 7,4′-dimethyl ether-5-glucoside (18.3-fold increase) standing out as markers of stability (Supplementary Table S5). In contrast, heat-dried powder (HDP) extract showed selective enhancement of certain metabolites, such as 6-hydroxyluteolin (27.4-fold increase in HDP vs. HD), but significant degradation of others like delphinidin 3-O-beta-D-sambubioside (0.16-fold decrease), suggesting thermal sensitivity (Supplementary Table S5). These findings underscore that freeze-drying is optimal for preserving broad-spectrum flavonoids, while heat drying may be suitable for targeting specific heat-stable antioxidants.

The transition from fresh samples to powder extracts (FSP vs. FS) led to remarkable upregulation of key compounds, including pedaliin (32.5-fold increase) and eriodictyol chalcone (6.7-fold increase), likely due to dehydration-induced concentration or inactivation of degradative enzymes (Supplementary Table S5). However, HDP vs. HD comparisons revealed that heat-drying powders amplified some flavonoids (e.g., gossypetin 3,4′-dimethyl ether, 53-fold increase) while degrading others (e.g., sophoranochromene, 0.06-fold decrease). Notably, FDP vs. FD (freeze-dried powder extract vs. freeze-dried whole flower) showed consistent upregulation of most flavonoids (e.g., tricin-7-O-acetylglucoside, 3.6-fold increase), suggesting powder extracting enhances extractability without major losses (Supplementary Table S5). This implies that powdered forms—especially freeze-dried—can intensify bioactive content, but processing methods must be tailored to avoid compound-specific degradation.

### 3.6 Antioxidant capacity of loquat flower extracts

**Figure 6:**
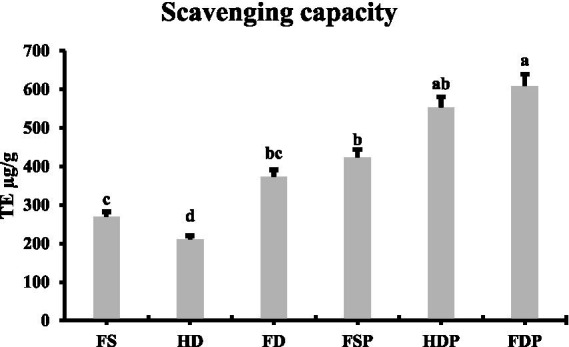
DPPH radical scavenging capacity of differently processed loquat flower samples. Column graph comparing the antioxidant activity expressed as μg Trolox equivalents per gram of fresh samples (FS), heat-dried (HD), freeze-dried (FD) loquat flowers and their corresponding powdered extracts fresh sample powder (FSP), heat drying powder (HDP), and freeze-drying powder (FDP). Values represent means ± standard deviation (*n* = 3). Different lowercase letters indicate significant differences between groups (*p* < 0.05 by LSD test).

The DPPH radical scavenging assay revealed significant differences in antioxidant capacity among processed loquat flower samples (Figure 6). FDP exhibited the highest antioxidant activity (608.83 μg TE/g), followed by HDP (552.89 μg TE/g) and fresh sample powder (FSP, 423.21 μg TE/g) (Figure 6). Among non-powdered samples, freeze-dried flowers (FD) showed superior antioxidant capacity (372.78 μg TE/g) compared to fresh (FS, 268.87 μg TE/g) and heat-dried (HD, 209.82 μg TE/g) samples (Figure 6). Notably, powdered extracts consistently outperformed their whole-flower counterparts, suggesting that hot-water extraction and subsequent freeze-drying enhance antioxidant bioavailability.

### 3.7 Practical implications for functional food and nutraceutical development

The data underscore that drying methods critically influence the phytochemical profile of loquat flower products. Freeze-drying appears optimal for retaining structurally complex flavonoids, making it suitable for high-value nutraceutical applications (Supplementary Table S4). In contrast, heat drying may be more appropriate for enhancing specific metabolites (e.g., 6-hydroxyluteolin, quercetagetin derivatives), which could be beneficial for antioxidant-rich ingredient formulations (Supplementary Table S4). However, the significant loss of anthocyanins like delphinidin 3-O-sambubioside in HDP suggests that thermal processing may compromise color-related bioactive properties (Supplementary Table S4). For manufacturers, selecting a drying method should depend on the target bioactive compounds—freeze-drying for maximal flavonoid preservation or heat drying for cost-effective production of select antioxidants. Freeze-drying powder extract is the gold standard for maximizing flavonoid diversity (e.g., cyanidin, 15.3-fold increase in FSP vs. FS), making it ideal for high-value nutraceuticals (Supplementary Table S5). Heat drying, while less universally protective, may be cost-effective for products targeting specific metabolites like 6-hydroxyluteolin or quercetagetin derivatives (Supplementary Table S5). The dramatic increases in FSP vs. FS suggest that even minimal processing (e.g., fresh samples powder extracting) can enhance certain bioactive, but long-term stability requires further study. For industry applications, hybrid approaches—such as mild heat drying followed by freeze-drying—could balance cost and nutrient retention.

The elevated antioxidant activity in freeze-dried powders (FDP) aligns with their higher retention of key flavonoids like violanthin and cyanidin, as identified in metabolomic analysis. While heat-dried powder (HDP) also demonstrated strong antioxidant capacity (likely due to thermally stable compounds like syringetin) its lower flavonoid diversity may limit broader health benefits. These findings suggest that freeze-dried loquat flower extracts are ideal for premium nutraceuticals requiring maximal antioxidant potency, whereas heat-dried powders offer a cost-effective alternative for products targeting specific bioactive metabolites.

## 4 Discussion

The current study provides compelling evidence that processing methods significantly influence the flavonoid profile and antioxidant potential of loquat flowers, with freeze-drying emerging as the superior technique for preserving bioactive compounds. Our findings align with previous research on maoberry fruits, where freeze-drying consistently outperformed heat-drying in retaining thermolabile phytochemicals (8, 9, 36). The remarkable preservation of cyanidin (1.66% in FD vs. 0.45% in HD) and violanthin (3.38% in FDP vs. 2.80% in HDP) in freeze-dried samples supports earlier observations that anthocyanins and flavonoid glycosides are particularly susceptible to thermal degradation (37–39). This thermal sensitivity explains why heat-dried samples showed fewer upregulated metabolites (16 in HD vs. FS) compared to freeze-dried counterparts (39 in FD vs. FS). The superior retention of these compounds in freeze-dried powders (FDP) further confirms that lyophilization, when combined with aqueous extraction, enhances the stability of polar flavonoids—a phenomenon previously reported in berry processing (9).

Interestingly, our results revealed that certain metabolites like methyl hesperidin (10.04% in HD) and syringetin (8.98% in HD) were more abundant in heat-dried samples, suggesting thermal processing may induce structural modifications or enhance extractability of specific compounds. This finding corroborates studies on citrus peels, where heat treatment increased hesperidin content through cell wall disruption (40–44). However, the dramatic reduction of heat-sensitive compounds like delphinidin 3-O-sambubioside (0.16-fold in HDP vs. FSP) underscores a critical trade-off—while heat drying may enhance some metabolites, it risks degrading others with proven health benefits. This dual effect aligns with observations in rosemary processing, where oven drying increased carnosic acid but reduced rosmarinic acid (45, 46). Our PCA and HCA analyses further reinforced these trends, showing clear separation between heat- and freeze-dried groups, consistent with metabolomic studies on other medicinal flowers (47, 48).

The transformation from fresh flowers to powdered extracts introduced another layer of complexity to our findings. The homogenization effect observed in powdered samples (FSP, HDP, FDP)—evidenced by tighter clustering in HCA—mirrors report on ginseng and turmeric processing, where milling and extraction reduced metabolite variability (49–51). Notably, freeze-dried powders (FDP) showed exceptional flavonoid enrichment (e.g., 58.1-fold increase in 5,7,2′,5′-tetrahydroxyflavanone), likely due to water removal concentrating bioactive compounds while minimizing oxidative damage—a phenomenon documented in oyster mushroom (*Pleurotus ostreatus*) processing (52). However, the significant downregulation of 5,7,2′,5′-tetrahydroxy-8,6′-dimethoxyflavone (from 23.85% in FS to 5.29% in FD) suggests some flavones may degrade during dehydration regardless of method, echoing concerns raised about flavonoid stability in prolonged processing (4).

The superior antioxidant capacity of freeze-dried loquat flower powder (FDP) aligns with its higher flavonoid content, particularly thermolabile compounds like cyanidin, consistent with studies on other plants (38). While heat-dried powder (HDP) showed unexpectedly strong activity, this likely reflects the formation of Maillard reaction products with antioxidant properties, as reported in heat-processed teas. The enhanced activity in powdered extracts versus whole flowers supports previous findings that processing improves bioactive compound extractability capacity (53–55). Freeze-drying offers maximal phytochemical preservation however, its higher operational costs (3–5 × energy consumption vs. conventional drying (8, 36)) may limit scalability for budget-sensitive products. As demonstrated in our study, heat drying remains viable for products targeting specific heat-stable compounds like syringetin or methyl hesperidin. This trade-off mirrors observations in maoberry processing, where freeze-drying preserved 92% anthocyanins versus 68% in oven-dried samples despite similar cost disparities (36).

For manufacturers seeking middle ground, hybrid approaches show promise. As evidenced in berry processing (56), sequential mild-heat drying (50–60°C) followed by freeze-drying reduced energy use by 35% while maintaining 88% flavonoid retention—a compelling balance for mid-range nutraceuticals. Such protocols could be particularly valuable for loquat flowers, given their similar thermolabile flavonoid profile. Future research should explore hybrid processing (e.g., mild heat drying followed by freeze-drying) or antioxidant pretreatment—strategies that have shown promise in preserving blueberries and medicinal herbs (56–60). Our study thus advances the optimization of loquat flower processing while highlighting the need for compound-specific approaches in functional food development. While we focused on processing-induced compositional changes (a critical first step for industrial optimization), we acknowledge the need for future *in vivo* validation, bioavailability assays, sensory properties, and long-term stability testing to bridge the gap to clinical or consumer applications. Our findings lay a foundational framework for such studies, with clear translational potential: the identified metabolites and processing protocols can guide targeted nutraceutical development. Future work should expand to hybrid drying techniques, sensory evaluation, and multi-origin sampling to enhance generalizability, building on this study’s systematic metabolomic and antioxidant profiling.

## 5 Conclusion

This study demonstrates that freeze-drying optimally preserves bioactive flavonoids in loquat flowers, retaining 39 upregulated metabolites versus fresh samples, while heat-drying degrades thermolabile compounds like cyanidin (6.62-fold reduction). Freeze-dried powder (FDP) showed superior antioxidant capacity (608.83 μg TE/g), correlating with enhanced flavonoid retention. For industrial applications, freeze-drying is ideal for premium nutraceuticals despite higher costs (~3–5 × energy vs. heat-drying), while heat-drying suits cost-conscious products targeting heat-stable metabolites (e.g., methyl hesperidin at 10.04%). A hybrid approach (mild heat + freeze-drying) may balance cost and quality, potentially reducing energy use by 30–40% while preserving 85–90% of key flavonoids. Future research should validate bioavailability, long-term stability, and hybrid processing scalability. These findings provide actionable insights for optimizing loquat flower processing in functional food and nutraceutical industries.

## Data Availability

The original contributions presented in the study are included in the article/Supplementary material, further inquiries can be directed to the corresponding authors.

## Footnotes

^1^ http://www.metware.cn/

^2^ https://sciex.com.cn/

^4^ http://www.omicshare.com/tools

^5^ https://www.bic.ac.cn/EVenn/#/

## References

[1] Dhiman A Suhag R Thakur D Gupta V Prabhakar PK. Current status of loquat (*Eriobotrya japonica* Lindl.): bioactive functions, preservation approaches, and processed products. Food Rev Int. (2022) 38:286–316. doi: 10.1080/87559129.2020.1866007

[2] Shah HMS Khan AS Singh Z Ayyub S. Postharvest biology and Technology of Loquat (*Eriobotrya japonica* Lindl.). Foods. (2023) 12:1329. doi: 10.3390/foods12061329, PMID: 36981255 PMC10048680

[3] Gao Y Xia W Shao P Wu W Chen H Fang X. Impact of thermal processing on dietary flavonoids. Curr Opin Food Sci. (2022) 48:100915. doi: 10.1016/j.cofs.2022.100915

[4] ElGamal R Song C Rayan AM Liu C Al-Rejaie S ElMasry G. Thermal degradation of bioactive compounds during drying process of horticultural and agronomic products: a comprehensive overview. Agronomy. (2023) 13:1580. doi: 10.3390/agronomy13061580

[5] Rezvankhah A Emam-Djomeh Z Askari G. Encapsulation and delivery of bioactive compounds using spray and freeze-drying techniques: a review. Dry Technol. (2020) 38:235–58. doi: 10.1080/07373937.2019.1653906

[6] Chaaban H Ioannou I Chebil L Slimane M Gérardin C Paris C. Effect of heat processing on thermal stability and antioxidant activity of six flavonoids. J Food Process Preserv. (2017) 41:e13203. doi: 10.1111/jfpp.13203

[7] Nguyen Q.V. Chuyen H. Van Processing of herbal tea from roselle (*Hibiscus sabdariffa* L.): effects of drying temperature and brewing conditions on total soluble solid, phenolic content, antioxidant capacity and sensory quality Beverages (2020) 6:2. doi: 10.3390/beverages6010002

[8] Gat Y Gawande P. Freeze-drying effect on nutrients and their stability In: Waghmare RB Kumar M Panesar PS, editors. Freeze drying of food products: fundamentals, processes and applications. Hoboken, New Jersey, USA: John Wiley & Sons Ltd. (2024). 179–201.

[9] Kittibunchakul S Temviriyanukul P Chaikham P Kemsawasd V. Effects of freeze drying and convective hot-air drying on predominant bioactive compounds, antioxidant potential and safe consumption of Maoberry fruits. LWT. (2023) 184:114992. doi: 10.1016/j.lwt.2023.114992

[10] Velazquez-Martinez V. Valles-Rosales D. Rodriguez-Uribe L. Holguin O. Quintero-Quiroz J. Reyes-Jáquezet D.. Antimicrobial, Shelf-Life Stability, and Effect of Maltodextrin and Gum Arabic on the Encapsulation Efficiency of Sugarcane Bagasse Bioactive Compounds. Foods. (2021). 10:116. doi: 10.3390/foods10010116 33429841 PMC7827221

[11] Sagar NA Pareek S Bhardwaj R Vyas N. Bioactive compounds of loquat (*Eriobotrya japonica* (Thunb.) L.) In: Murthy H Bapat V, editors. Bioactive compounds in underutilized fruits and nuts. Cham: Springer (2020). 123–43.

[12] Bermejo A Llosá MJ Cano A. Analysis of bioactive compounds in seven Citrus cultivars. Food Sci Technol Int. (2011) 17:55–62. doi: 10.1177/1082013210368556, PMID: 21364046

[13] Kerdsomboon K Chumsawat W Auesukaree C. Effects of *Moringa oleifera* leaf extracts and its bioactive compound Gallic acid on reducing toxicities of heavy metals and metalloid in *Saccharomyces cerevisiae*. Chemosphere. (2021) 270:128659. doi: 10.1016/j.chemosphere.2020.128659, PMID: 33757277

[14] Rao MJ Duan M Wang J Han S Ma L Mo X. Transcriptomic and widely targeted metabolomic approach identified diverse group of bioactive compounds, antiradical activities, and their associated genes in six sugarcane varieties. Antioxidants. (2022) 11:1319. doi: 10.3390/antiox11071319, PMID: 35883810 PMC9311902

[15] Rao MJ Tahir Ul Qamar M Wang D Ali Q Ma L Han S. A high-throughput lipidomics and transcriptomic approach reveals novel compounds from sugarcane linked with promising therapeutic potential against COVID-19. Front Nutr. (2022) 9:988249. doi: 10.3389/fnut.2022.988249, PMID: 36118771 PMC9480494

[16] Jang EB Ho T-T Park SY. Effect of light quality and tissue origin on phenolic compound accumulation and antioxidant activity in *Camellia japonica* Calli. In Vitro Cell Dev Biol Plant. (2020) 56:567–77. doi: 10.1007/s11627-020-10121-9

[17] Gao H Wang Q Lai C Jiang F. Optimized formulation of a chewable candy containing loquat flower tea. Fujian J Agric Sci. (2021) 36:964–71.

[18] Lu ZM Wu WX Zhang ZL. Effect of flower and fruit thinning on yield and quality of loquat in northern margin of loquat distribution area in China. Proc III Int Symp Loquat. (2010) 887:165–9.

[19] Zheng M Xia Q Lu S. Study on drying methods and their influences on effective components of loquat flower tea. LWT Food Sci Technol. (2015) 63:14–20. doi: 10.1016/j.lwt.2015.03.090

[20] Rao MJ Duan M Zhou C Jiao J Cheng P Yang L. Antioxidant defense system in plants: reactive oxygen species production, signaling, and scavenging during abiotic stress-induced oxidative damage. Horticulturae. (2025) 11:477. doi: 10.3390/horticulturae11050477

[21] Duan M Bao L Eman M Han D Zhang Y Zheng B. The ectopic expression of the MpDIR1(t) gene enhances the response of plants from *Arabidopsis thaliana* to biotic stress by regulating the defense genes and antioxidant flavonoids. Plants. (2024) 13:2692. doi: 10.3390/plants13192692, PMID: 39409562 PMC11478391

[22] Zhu G Guo H Huang Y Wu C Zhang X. Eriodictyol, a plant flavonoid, attenuates LPS-induced acute lung injury through its antioxidative and anti-inflammatory activity. Exp Ther Med. (2015) 10:2259–66. 26668626 10.3892/etm.2015.2827 PMC4665393

[23] Shen N Wang T Gan Q Liu S Wang L Jin B. Plant flavonoids: classification, distribution, biosynthesis, and antioxidant activity. Food Chem. (2022) 383:132531. doi: 10.1016/j.foodchem.2022.132531, PMID: 35413752

[24] Rao MJ Wang H Lei H Zhang H Duan X Bao L. LC-MS/MS-based metabolomic study provides insights into altitude-dependent variations in flavonoid profiles of strawberries. Front Plant Sci. (2025) 15:1527212. doi: 10.3389/fpls.2024.1527212, PMID: 39840353 PMC11746042

[25] Williams RJ Spencer JPE Rice-Evans C. Flavonoids: antioxidants or Signalling molecules? Free Radic Biol Med. (2004) 36:838–49. doi: 10.1016/j.freeradbiomed.2004.01.001, PMID: 15019969

[26] Yu J Wang L Walzem RL Miller EG Pike LM Patil BS. Antioxidant activity of Citrus Limonoids, flavonoids, and Coumarins. J Agric Food Chem. (2005) 53:2009–14. doi: 10.1021/jf0484632, PMID: 15769128

[27] Dini C Zaro MJ Viña SZ. Bioactivity and functionality of anthocyanins: a review. Curr Bioact Compd. (2019) 15:507–23. doi: 10.2174/1573407214666180821115312

[28] Silva A Silva V Igrejas G Aires A Falco V Valentão P. Phenolic compounds classification and their distribution in winemaking by-products. Eur Food Res Technol. (2023) 249:207–39. doi: 10.1007/s00217-022-04163-z

[29] Rao MJ Duan M Shad MA Aslam MZ Wang J Wang L. Widely targeted LC-MS/MS approach provides insights into variations in bioactive flavonoid compounds and their antioxidant activities in green, red, and purple sugarcane. LWT Food Sci Technol. (2024) 209:116792. doi: 10.1016/j.lwt.2024.116792

[30] Nile SH Keum YS Nile AS Jalde SS Patel RV. Antioxidant, anti-inflammatory, and enzyme inhibitory activity of natural plant flavonoids and their synthesized derivatives. J Biochem Mol Toxicol. (2018) 32:e22002. doi: 10.1002/jbt.22002, PMID: 28972678

[31] Borges Bubols G da Rocha Vianna D Medina-Remon A von Poser G Maria Lamuela-Raventos R Lucia Eifler-Lima V. The antioxidant activity of Coumarins and flavonoids. Mini Rev Med Chem. (2013) 13:318–34. 22876957 10.2174/138955713804999775

[32] Rao MJ Wang L Ahmad U Ahmad MH Hussain S. Citrus metabolic and antioxidant responses to high light stress In: Hussain S Khalid MF Ali MA Ahmed N Hasanuzzaman M Ahmad S, editors. Citrus production - technological advancements and adaptation to changing climate. Boca Raton, FL, USA: CRC Press (2022). 167–79.

[33] Chen W Gong L Guo Z Wang W Zhang H Liu X. A novel integrated method for large-scale detection, identification, and quantification of widely targeted metabolites: application in the study of Rice metabolomics. Mol Plant. (2013) 6:1769–80. doi: 10.1093/mp/sst080, PMID: 23702596

[34] Dudonne S Vitrac X Coutiere P Woillez M Mérillon J-M. Comparative study of antioxidant properties and Total phenolic content of 30 plant extracts of industrial interest using DPPH, ABTS, FRAP, SOD, and ORAC assays. J Agric Food Chem. (2009) 57:1768–74. doi: 10.1021/jf803011r, PMID: 19199445

[35] Mu H Chen J Huang W Huang G Deng M Hong S. Omicshare tools: a zero-code interactive online platform for biological data analysis and visualization. iMeta. (2024) 3:e228. doi: 10.1002/imt2.228, PMID: 39429881 PMC11488081

[36] Chumroenphat T Somboonwatthanakul I Saensouk S Siriamornpun S. Changes in Curcuminoids and chemical components of turmeric (*Curcuma longa* L.) under freeze-drying and low-temperature drying methods. Food Chem. (2021) 339:128121. doi: 10.1016/j.foodchem.2020.128121, PMID: 33152891

[37] Zhao Y-W Wang C-K Huang X-Y Hu D-G. Anthocyanin stability and degradation in plants. Plant Signal Behav. (2021) 16:1987767. doi: 10.1080/15592324.2021.1987767, PMID: 34686106 PMC9208790

[38] Enaru B Drețcanu G Pop TD Stǎnilǎ A Diaconeasa Z. Anthocyanins: factors affecting their stability and degradation. Antioxidants. (2021) 10:1967. doi: 10.3390/antiox10121967, PMID: 34943070 PMC8750456

[39] Ioannou I Chekir L Ghoul M. Effect of the processing temperature on the degradation of food flavonoids: kinetic and calorimetric studies on model solutions. J Food Eng Technol. (2019) 8:91–102. doi: 10.32732/jfet.2019.8.2.91

[40] Shetty SB Mahin-Syed-Ismail P Varghese S Thomas-George B Kandathil-Thajuraj P Baby D. Antimicrobial effects of *Citrus sinensis* peel extracts against dental caries bacteria: an in vitro study. J Clin Exp Dent. (2016) 8:e71–7. doi: 10.4317/jced.52493, PMID: 26855710 PMC4739372

[41] Chen M Yang D Liu S. Effects of drying temperature on the flavonoid, phenolic acid and antioxidative capacities of the methanol extract of (*Citrus sinensis* (L.) Osbeck) peels. Int J Food Sci Technol. (2011) 46:1179–85.

[42] Mencherini T Campone L Piccinelli AL Garcia Mesa M Sánchez DM Aquino RP. HPLC-PDA-MS and NMR characterization of a hydroalcoholic extract of *Citrus aurantium* L. var. amara peel with antiedematogenic activity. J Agric Food Chem. (2013) 61:1686–93. doi: 10.1021/jf302815t, PMID: 22957519

[43] Singh J Sood S Muthuraman A. In-vitro evaluation of bioactive compounds, anti-oxidant, lipid peroxidation and lipoxygenase inhibitory potential of *Citrus karna* L. peel extract. J Food Sci Technol. (2014) 51:67–74. doi: 10.1007/s13197-011-0479-9, PMID: 24426049 PMC3857418

[44] Zhang L Ling W Yan Z Liang Y Guo C Ouyang Z. Effects of storage conditions and heat treatment on the hesperidin concentration in Newhall navel orange (*Citrus sinensis* Osbeck cv. Newhall) juice. J Food Compos Anal. (2020) 85:103338. doi: 10.1016/j.jfca.2019.103338

[45] An K Zhao D Wang Z Wu J Xu Y Xiao G. Comparison of different drying methods on Chinese ginger (*Zingiber Officinale* roscoe): changes in volatiles, chemical profile, antioxidant properties, and microstructure. Food Chem. (2016) 197:1292–300. doi: 10.1016/j.foodchem.2015.11.033, PMID: 26675871

[46] Chua LYW Chong CH Chua BL Figiel A. Influence of drying methods on the antibacterial, antioxidant and essential oil volatile composition of herbs: a review. Food Bioprocess Technol. (2019) 12:450–76. doi: 10.1007/s11947-018-2227-x

[47] Wu Q Yan Q Jiang L Chen C Huang X Zhu X. Metabolomics analysis reveals metabolite changes during freeze-drying and oven-drying of *Angelica dahurica*. Sci Rep. (2023) 13:6022. doi: 10.1038/s41598-023-32402-0, PMID: 37055447 PMC10102171

[48] ElNaker NA Daou M Ochsenkühn MA Amin SA Yousef AF Yousef LF. A metabolomics approach to evaluate the effect of Lyophilization versus oven drying on the chemical composition of plant extracts. Sci Rep. (2021) 11:22679. doi: 10.1038/s41598-021-02158-6, PMID: 34811431 PMC8608909

[49] Salem MA El-Shiekh RA Fernie AR Alseekh S Zayed A. Metabolomics-based profiling for quality assessment and revealing the impact of drying of turmeric (*Curcuma longa* L.). Sci Rep. (2022) 12:10288. doi: 10.1038/s41598-022-13882-y, PMID: 35717541 PMC9206664

[50] Jeong H Park DH Seo HG Choi M-J Cho Y. Effect of roasting time and cryogenic milling on the physicochemical characteristics of dried ginseng powder. Foods. (2020) 9:223. doi: 10.3390/foods9020223, PMID: 32093227 PMC7073925

[51] Sun M Li K Li X Li J Men L Lu M. Phytochemical properties and Antioxidative activities of ginseng superfine powders prepared by wet grinding method with high-pressure homogenization. Powder Technol. (2025) 451:120448. doi: 10.1016/j.powtec.2024.120448

[52] Ucar TM Karadag A. The effects of vacuum and freeze-drying on the physicochemical properties and in vitro digestibility of Phenolics in oyster mushroom (*Pleurotus ostreatus*). J Food Meas Charact. (2019) 13:2298–309. doi: 10.1007/s11694-019-00149-w

[53] Sun M-J Chiang Y-C Hou C-Y Ciou J-Y. Effect of combining moisture-assisted drying on lemon (*Citrus limon* (L.) Brum.): physicochemical properties, antioxidant capacities, and sensory evaluations. Food Bioprocess Technol. (2025) 18:508–18.

[54] Wei L Shaoyun W Shutao L Jianwu Z Lijing K Pingfan R. Increase in the free radical scavenging capability of bitter gourd by a heat-drying process. Food Funct. (2013) 4:1850–5. doi: 10.1039/c3fo60169b, PMID: 24192975

[55] Abbaspour-Gilandeh Y Kaveh M Fatemi H Aziz M. Combined hot air, microwave, and infrared drying of hawthorn fruit: effects of ultrasonic pretreatment on drying time, energy, qualitative, and bioactive compounds’ properties. Foods. (2021) 10:1006. doi: 10.3390/foods10051006, PMID: 34064476 PMC8147953

[56] Sun Y Zhang M Mujumdar A. Berry drying: mechanism, pretreatment, drying technology, nutrient preservation, and mathematical models. Food Eng Rev. (2019) 11:61–77. doi: 10.1007/s12393-019-9188-3

[57] Athira VA Gokulvel E Nandhu Lal AM Venugopalan VV Rajkumar Venkatesh T. Advances in drying techniques for retention of antioxidants in agro produces. Crit Rev Food Sci Nutr. (2023) 63:10849–65. 35653131 10.1080/10408398.2022.2082371

[58] Yu ZM Jun LJ Jun FJ Min LS. Effect of drying methods on sensory qualities of loquat flower tea and its toxicological evaluation. Food Sci. (2018) 39:17.

[59] Rao MJ Zheng B. The role of polyphenols in abiotic stress tolerance and their antioxidant properties to scavenge reactive oxygen species and free radicals. Antioxidants. (2025) 14:74. doi: 10.3390/antiox14010074, PMID: 39857408 PMC11761259

[60] Rao MJ Duan M Ikram M Zheng B. ROS Regulation and Antioxidant Responses in Plants Under Air Pollution: Molecular Signaling, Metabolic Adaptation, and Biotechnological Solutions. Antioxid. (2025) 14:907. doi: 10.3390/antiox14080907 PMC12383030 40867806

[61] Chong J Xia J. MetaboAnalystR: an R package for flexible and reproducible analysis of metabolomics data. Bioinform. (2018) 34, 4313–4314. 10.1093/bioinformatics/bty528 PMC6289126 29955821

[62] Colquhoun D. An investigation of the false discovery rate and the misinterpretation of p-values. R Soc Open Sci. (2014) 1, 140216. 26064558 10.1098/rsos.140216 PMC4448847

[63] Fraga CG Clowers BH Moore RJ Zink EM. Signature-discovery approach for sample matching of a nerve-agent precursor using liquid chromatography− mass spectrometry, XCMS, and chemometrics. Anal Chem (2010) 82, 4165–4173. 20405949 10.1021/ac1003568

[64] Thévenot EA Roux A Xu Y Ezan E Junot C. Analysis of the human adult urinary metabolome variations with age, body mass index, and gender by implementing a comprehensive workflow for univariate and OPLS statistical analyses. J Proteome Res (2015) 14, 3322–3335. 26088811 10.1021/acs.jproteome.5b00354

